# Non-invasive and label-free 3D-visualization shows *in vivo* oligomerization of the staphylococcal alkaline shock protein 23 (Asp23)

**DOI:** 10.1038/s41598-019-56907-9

**Published:** 2020-01-10

**Authors:** Inga Petersen, Rabea Schlüter, Katharina J. Hoff, Volkmar Liebscher, Gert Bange, Katharina Riedel, Jan Pané-Farré

**Affiliations:** 1 University of Greifswald, Institute of Microbiology, Felix-Hausdorff-Str. 8, 17489 Greifswald, Germany; 2 University of Greifswald, Imaging Center of the Department of Biology, Friedrich-Ludwig-Jahn-Str. 15, 17489 Greifswald, Germany; 3 University of Greifswald, Institute of Mathematics and Computer Science, Walther-Rathenau-Str. 47, 17489 Greifswald, Germany; 4 Center for Functional Genomics of Microbes, Felix-Hausdorff-Str. 8, 17489 Greifswald, Germany; 5 Philipps-University Marburg, SYNMIKRO Research Center and Department of Chemistry, Hans-Meerwein-Strasse 6, C07, 35043 Marburg, Germany

**Keywords:** 3-D reconstruction, Cellular microbiology

## Abstract

Fluorescence-tags, commonly used to visualize the spatial distribution of proteins within cells, can influence the localization of the tagged proteins by affecting their stability, interaction with other proteins or the induction of oligomerization artifacts. To circumvent these obstacles, a protocol was developed to generate 50 nm thick serial sections suitable for immunogold labeling and subsequent reconstruction of the spatial distribution of immuno-labeled native proteins within individual bacterial cells. Applying this method, we show a cellular distribution of the staphylococcal alkaline shock protein 23 (Asp23), which is compatible with filament formation, a property of Asp23 that we also demonstrate *in vitro*.

## Introduction

Introduction of fluorescently labeled protein tags, such as green-fluorescent protein (GFP), and the development of super-resolution fluorescence microscopy techniques have led to a renaissance of microbial cell biology appreciating the remarkably high degree of spatial-temporal organization of the bacterial cell physiology^1–3^.

However, fluorescently labeled proteins can exhibit altered or destroyed cellular functionalities, as previously reported, for instance, for the rod-shape determining protein MreB. Using yellow fluorescence protein (YFP) tagged MreB, it was initially suggested that MreB forms a continuous, cell-spanning helical filament running along the inner membrane of the cell^4^. A reevaluation of these conclusions using electron cryotomography showed that the cell spanning MreB helix is an artifact introduced by the YFP-tag^5^. Intriguingly, helical localization patterns were reported for many other fluorescently tagged bacterial proteins (e.g. chemoreceptors, secretion proteins, RNase E, and the chromosome partitioning protein SetB) but often the questions remains how well these observations reflect the true situation^6^.

Therefore, label-free methods are critically required in order to validate findings obtained by this commonly used method. One way to achieve this goal is the detection of proteins by immunofluorescence. This approach typically requires permeabilization or removal of the bacterial cell envelope in order to allow antibody penetration into bacterial cells. However, this experimental step provides a major source of artifact generation because removal of the cell wall significantly affects the integrity and structure of the cell. To circumvent this problem, we have developed a non-invasive protocol enabling the three-dimensional (3D-) visualization of proteins in serial sections of bacterial cells by immunofluorescence microscopy or immunogold labeling envisioned by electron microscopy. So far, serial sections electron microscopy (ssEM) of tissues and organs has been successfully used to, for example, reconstruct the 3D ultrastructure of the nervous system from higher organisms such as *Caenorhabditis elegans*, *Drosophila* and mammals^7^. However, at present no ssEM protocol was available to investigate protein localization in entire bacterial cells.

Therefore, to challenge the feasibility of this approach, we used the *Staphylococcus aureus* alkaline shock protein 23 (Asp23) as a model to gain a deeper insight into the spatial organization of Asp23 within the cell. Asp23 is the eponym of the poorly investigated Asp23 protein family (also termed DUF322, PF03780 or Gls24 family). Members of this protein family are exclusively present in Gram-positive bacteria^8^, where they are functionally linked to lipid metabolism (*Bacillus subtilis*)^9^, survival at low pH and during nutrient limitation (*Streptococcus agalactiae*)^10^, the bile stress response (*Enterococcus faecium*)^11^, cell morphology control (*Enterococcus faecalis*)^12^ and nutrient sensing (*Streptococcus pneumoniae*)^13^. However, the precise mechanisms underlying these functions remain to be elucidated.

**Figure 1:**
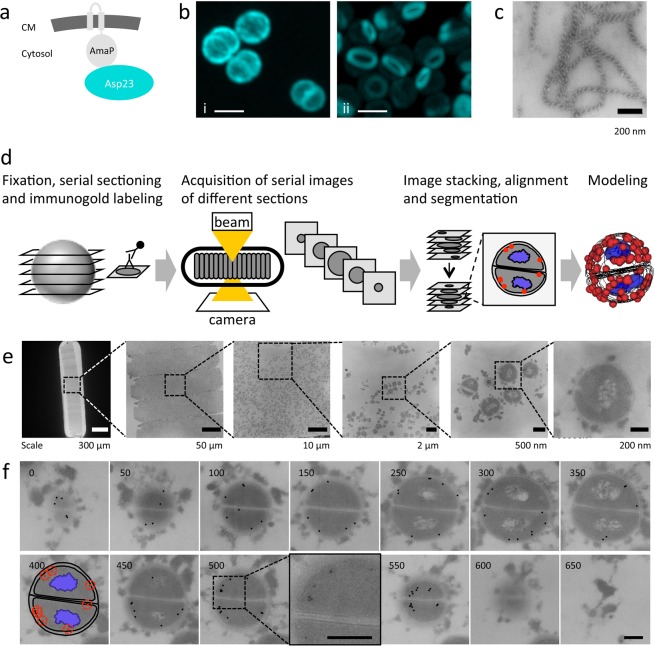
The *S. aureus* Asp23 protein and the serial section microscopy workflow. (**a**) Scheme of Asp23 membrane localization. AmaP is a small protein encoded within the same operon as Asp23. CM, cytoplasmic membrane. (**b**) Cellular localization of Cerulean-tagged Asp23 by fluorescence microscopy. In the wild type (i) Asp23 appears to be evenly distributed below the cell membrane. In the *amaP* mutant (ii), Asp23-Cer oligomerizes to ring shaped structures. Scale bar, 1 µm. (**c**) Purified Asp23 forms long telephone cord-like structures *in vitro*. Scale bar, 200 nm. (**d**) Serial section microscopy work flow. After fixation and resin embedding, cells are sectioned and subsequently labeled with the primary and secondary antibody for antigen detection. For fluorescence microscopy, a fluorescence conjugated antibody is used, while for electron microscopy the secondary antibody is linked to a gold particle. Following serial image acquisition of different sections, the software program Reconstruct is used to stack, align and segment the images to generate the final model. (**e**) Zoom in of an EM grid showing serial section of *S. aureus*. Scale bars from left to right micrograph as indicated below images. (**f**) Example of an entire single cell followed over 13 consecutive sections of 50 nm thickness. Segmentation is shown for the 400 nm plane. Gold particles were highlighted for better visualization in sections showing entire cells. Original contrast is shown in the 500 nm plane zoom in. Scale bar, 200 nm.

For the *S. aureus* Asp23 protein a critical function in cell envelope homeostasis was shown^8^. Asp23 attaches to the inner side of the cytoplasmic membrane through its interaction with the membrane protein AmaP (Asp23 membrane anchoring protein) (Fig. 1a) and deletion of either *asp23* or *amaP* leads to strong induction of the cell wall stress response^8^. Interestingly, aligning with its membrane localization properties and cell envelope related function, it was recently reported that loss of Asp23 leads to increased resistance towards the cell membrane-targeting antibiotic daptomycin^14^. The molecular function of Asp23, however, is still unclear. Using our serial section electron microscopy and immune-labeling protocol, we show a distribution pattern of Asp23 compatible with the formation of Asp23 filaments, a property of Asp23 that we also demonstrate *in vitro*. These results provide a framework for future investigations analyzing the relationship between Asp23 oligomer formation and function.

## Results and Discussion

Classical confocal fluorescence microscopy employing Asp23 fluorescently-labeled with Cerulean (Asp23-Cer) showed an even co-localization of Asp23-Cer with the membrane^8^. In the present study, we observed that in the absence of its membrane interaction partner AmaP, Asp23-Cer formed prominent ring-like structures within the cytoplasm, but not at the membrane (Fig. 1b). This finding suggested that Asp23 can oligomerize and form filaments under *in vivo* conditions.

However, we found that the *in vivo* formation of Asp23 filaments can also be drastically reduced by the introduction of an A206K amino acid substitution into the Cerulean-tag, which is known to disrupt the ability of fluorescent proteins to dimerize^15^ (Supplementary Fig. S1). This observation raises the question to which extent the fluorescent label alters the functional properties of Asp23, and demands an alternative method to investigate the sub-cellular architecture of Asp23 in a label-free manner. The ability of Asp23 to form filamentous structures is also supported *in vitro* with purified Asp23, which appears in extended telephone cord-like structures (Fig. 1c).

To clarify whether Asp23 does form filaments *in vivo* in the absence of a tag and to visualize the 3D-distribution of Asp23 in *S. aureus* cells, serial sections of *S. aureus* wild type and *amaP* mutant cells were probed with an antibody specific to Asp23. After immuno-labeling of Asp23, bound antibodies were visualized either by fluorescence microscopy, or by immunogold labeling combined with electron microscopy. Using the freely available software Reconstruct^16^, the fluorescence and electron micrographs were processed to reconstruct the Asp23 3D distribution within individual cells (Figs. 1d–f, S2 and S3).

**Figure 2:**
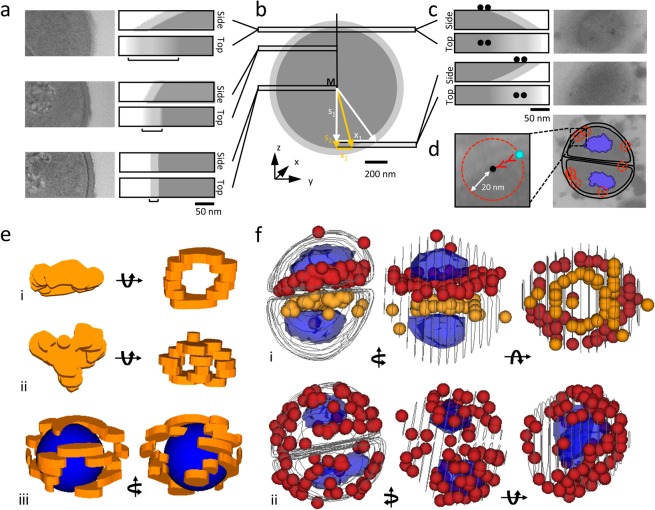
Analysis of Asp23 distribution. Section plane affects the appearance of cellular structures in electron micrographs. (**a**) With an increasing section angle across the cell envelope the cell wall appears progressively blurred. Original images and schemes showing the degree of cell wall blur (**b)**. The average position of the cell wall can be estimated using the extent of cell wall blurring and the distance of the section plane from middle of the cell (see Supplementary Information for details). (**c**) Original images and schemes showing that orientation of cell sections during immunodetection of antigen and (**d)** the linkage error affects the apparent localization of the antigen in electron micrographs. (**e**) 3D reconstruction of Asp23 distribution from immunofluorescence signals, (i and ii) *amaP* mutant and (iii) wild type. Volumes of Asp23 localization are shown with borders defined by the lateral fluorescence signal and the thickness of the section. For a better 3D visualization of Asp23 in the wild type, a blue sphere was drawn filling the cell lumen. (**f**) 3D reconstruction based on ssEM, orange and red spheres show the distribution of immunogold labeled Asp23 molecules, taking into account a linkage error of 40 nm, (i) *amaP* mutant and (ii) wild type. Blue elements mark the position of nucleoids. Lines indicate the position of the cell wall/cell membrane interface as deduced from the electron micrographs.

We found that the minimal section thickness suitable for immunofluorescence was 100 nm (Supplementary Fig. S4), while immunogold labeling and subsequent electron microscopy were compatible with sections of 50 nm thickness. It became evident that the section plane significantly affects the appearance of cellular structures when imaging objects as small as bacteria by electron microscopy. This is particularly obvious with the cell wall. Approaching the bottom or the top of the cell, sections will cut through the cell wall at a steadily increasing angle with the consequence of decreasing the cell wall signal-to-noise ratio (Fig. 2a). In this case, simple calculations can help to guide the positioning of the cell wall borders during image segmentation (Fig. 2b). Orientation of the section during immune-detection of antigens also affected the apparent localization of the antigen. For instance, on sections close to the top of the cell, gold-labeling on the upper side produced images where the protein appeared to be localized within the wall structure, while gold-labeling on the lower side suggested a cytoplasmatic localization (Fig. 2c). Thus, the positioning of cell borders must be carefully considered before volume reconstruction to avoid protein mislocalization in the final model.

3D reconstructions from serial sections showed a distribution of Asp23 in the *amaP* mutant compatible with the formation of ring-like polymers as suggested by widefield visualization of Cerulean-tagged Asp23 (Fig. 2e(i,ii),f(i)). The Asp23 distribution in wild type cells confirmed the observation that during cell division Asp23 does not localize to the septum and further revealed that Asp23 is not distributed evenly at the cell membrane, as initially suggested by fluorescence microscopy of Asp23-Cer (Fig. 2e (iii),f(ii)). The distribution pattern of Asp23 in the *amaP* mutant (ring-like) and in the wild type (surface associated) is supported by a statistical analysis using Ripleys K-function^17^ (Supplementary Figs. S5–S7).

The lateral resolution in our reconstructions is determined by the linkage error introduced by the probes used for immune detection and could be further improved if whole antibodies were substituted with e.g. Fab-fragments (Fig. 2d). Using antibodies of different specificity and labeled with gold particles of various size, colocalization experiments could also be carried out. Since only the antigens on the surface of the section are accessible to the antibody, a partially patchy distribution for the targeted protein might result. Finally, this method is limited to fixed cells and the analysis of localization dynamics is therefore only possible at discrete time points. However, while these obstacles may impede the demonstration of finer distribution patterns, distinct arrangements like rings in the case of Asp23 can be reconstructed with reasonable precision (Supplementary Movies S1–S4) The protocol developed here and our findings on spatial Asp23 distribution provide the framework to further evaluate if and how oligomerization affects Asp23 membrane localization and function in the cell envelope stress response.

## Conclusion

The procedure outlined here serves as an easily adoptable tool to visualize the 3D-distribution of proteins within objects as small as bacterial cells. It can be used to validate information obtained by fluorescently labeled proteins and is entirely suited to even basic EM equipment in order to directly monitor protein localization in bacteria in a label-free manner. Easy to use software-packages, such as Reconstruct^16^, Free-D^18^ and TrakEM2^19^ for image reconstruction are freely available, making the entire method relatively inexpensive. Taken together, the high resolution of the electron microscope in combination with the reconstruction of protein distribution from immunogold-labeled serial sections produces valuable and important information to complement and extend the data obtained with fluorescently tagged proteins in single bacterial cells or the organelles of higher organisms. Together with the finding that Asp23 also forms filaments *in vitro*, assays are now available to further investigated the function-structure relationship of this prominent member of the Asp23 protein family.

## Methods

### Sample preparation for serial sectioning and immuno-labeling

The following points should be considered as critical for the experimental success: (i) for *S. aureus*, the immunoglobulin binding surface protein A (*spa*) gene, should be deleted to avoid non-specific antibody binding, and (ii) the adequate aldehyde concentration required for cell fixation but not interfering with antibody efficiency has to be determined experimentally.

After 6 h of cultivation in LB medium, 1% glutaraldehyde (Sigma-Aldrich), 4% paraformaldehyde (Science Services GmbH, Germany), and 0.2% picric acid (AppliChem GmbH, Germany) were added to the growth medium and cells were fixed for 5 min at 40 °C by using a microwave processor for laboratory use (H2500 Microwave Processor, Energy Beam Sciences Inc. East Granby, Connecticut, USA), and then for 30 min at room temperature. Finally, samples were stored over night at 4 °C until further processing.

Subsequent to embedding in 2% low gelling agarose (VWR), cells were washed in buffer (100 mM cacodylate buffer [pH 7.4], 0.09 M sucrose, 1 mM CaCl_2_) twice for 30 min, treated with 0.1% tannic acid (Sigma-Aldrich) in buffer for 30 min and washed with buffer twice for 30 min at room temperature. After dehydration in graded series of ethanol (30%, 50%, 70%, 90%, 100% for 30 min each step on ice; Carl Roth GmbH) the material was infused with the acryl resin LR White (Plano GmbH). For this, 1 part 100% ethanol (VWR) was mixed with 1 part LR White and stored at 4 °C overnight. Subsequently 1 part 100% ethanol was mixed with 2 parts LR White for 2 h on ice followed by infiltration with pure resin for 6 h on ice, resin changing and storage at 4 °C overnight. Finally, samples were infused with pure resin at room temperature in sealed gelatine capsules (Plano GmbH). The resin was polymerized for 48 h at 60 °C. The resulting specimen block was then trimmed (Reichert Ultratrim, Leica UK Ltd, Milton Keynes, UK). Ultrathin serial sections (approximately 50 nm) were obtained by adding glue (pattex glue and xylol mixed in a ratio 1:1) on one side of the trimmed area and cut on an ultramicrotome (Reichert Ultracut, Leica UK Ltd) by using 8° angle of the knife and a cutting speed set to 0.6 mm/s. The sections were picked up with a nickel slot grid (2 × 0.5 mm; G2550N; Plano GmbH, Germany) and transferred onto a second, 1% pioloform-coated (Plano GmbH) nickel slot grid (2 × 0.5 mm) prior to immunogold labeling.

For immunofluorescence labeling, ultrathin serial sections (approximately 100 nm) were transferred onto poly-L-lysine (Sigma-Aldrich; 1:10 diluted with deionized water) coated 10-mm-diameter high precision coverslips (Paul Marienfeld GmbH, Germany) by using a nickel slot grid (2 × 0.5 mm; Plano GmbH). The coverslips have been placed on a 40 °C hot heating plate before transferring the sections. After evaporation of the liquid, the nickel slot grid was removed and the coverslips with the sections on top were stored at room temperature (RT) in a desiccator in the dark.

### Immunofluorescence labeling

The flotation method was used for immunofluorescence labeling: the coverslips were placed with the sections face down on the droplets of washing or antibody solution at RT. All droplets were placed successively on a flat sheet of parafilm. Droplets of 50 µl were used for all washing steps, and 30 µl droplets were used for incubating with antibodies. During the antibody incubation a humidity chamber was simulated by putting damp tissue paper around the parafilm before covering it with a large glass plate. When switching between different buffers or antibodies, excess liquid was wicked off by touching the edge of the coverslip with filter paper.

The specimens were washed three times for in total 5 min with Tris-buffered saline (TBS; 10 mM Tris, 150 mM NaCl, 20 mM NaN_3_; pH 8.0) and then rinsed twice for 10 min each time with glycine-TBS (50 mM glycine in TBS; filtered through a 0.2 µm syringe attachment filter [Carl Roth GmbH]) and 15 min with 5% goat serum (v/v; Invitrogen) in incubation buffer (0.2% gelatine [w/v; VWR], 1% skim milk powder [w/v; VWR], 0.1% Tween 20 in TBS). After blocking, specimens were washed three times for 5 min each time with incubation buffer and then incubated with the anti-Asp23 primary antibody, diluted to 1:50 in incubation buffer, for 1 h in the dark. Afterwards, specimens were washed six times for 2 min each time with incubation buffer, and then incubated with the secondary antibody conjugated with Alexa Fluor 546 (goat anti-rabbit IgG (H + L), Invitrogen Karlsruhe, Germany), diluted to 1:1000 in phosphate buffered saline (PBS; 137 mM NaCl, 2,7 mM KCl, 1,4 mM KH_2_PO_4_, 10 mM Na_2_HPO_4_ × 2 H_2_O; pH 7.4), for 1 h in the dark. After intensive washing steps, four times for 5 min each time with incubation buffer, three times for 5 min each time with PBS, and five times for 2 min each time with deionized water, the excess liquid was removed with filter paper and specimens were allowed to dry at RT. Finally, coverslips were stored in a desiccator in the dark at RT until they were imaged.

### Fluorescence microscopy of serial sections

For fluorescence microscopy of immunofluorescence-labeled thin sections, coverslips carrying the sections were embedded in >98% glycerol (Carl Roth GmbH) on glass slides (Marienfeld Superior). Imaging was carried out with a Zeiss Axio Imager.M2 (Carl Zeiss Microscopy GmbH, Oberkochen, Germany) equipped with a 100×/NA 1.3 Oil Neofluar objective, and Alexa Fluor 546 fluorescence signals were detected with 559–85 nm excitation filter and 600–90 nm emission filter. For each section, a z-stack of 13 images with a step size of 150 nm was acquired. Subsequently, deconvolution was carried out with Huygens Professional (Scientific Volume Imaging B.V) using 50 iterations with a theoretically calculated PSF, and automatic background correction. Maximum intensity projections were generated for each z-stack using ImageJ (v1.52b, Wayne Rasband, National Institutes of Health). The alignment of serial sections was carried out using Delta2D (v4.6, Decodon GmbH) with ‘exact’ warping settings and the manual spot selection tool. Further image processing was carried out with ImageJ (v1.52b, Wayne Rasband, National Institutes of Health).

### Immunogold labeling

The immunogold labeling was carried out according to the method described for immunofluorescence labeling regarding the general procedure with sections located on single slot grids (see Sample preparation).

The specimens were washed three times for 5 min in total with TBS, rinsed two times for 10 min each time with glycine-TBS, and 15 min with 5% (v/v) goat serum in incubation buffer. After blocking, specimens were washed three times for 5 min each time with incubation buffer and then incubated with the anti-Asp23 primary antibody, diluted to 1:50 in incubation buffer, for 1 h in the dark. Afterwards, specimens were washed six times for 2 min each time with incubation buffer, incubated for 1 h in the dark with the secondary antibody goat anti-rabbit 5-nm-diameter gold conjugates (Sigma-Aldrich), diluted 1:100 in incubation buffer, then rinsed five times for 4 min each time with incubation buffer, and three times for 5 min each time with PBS. Finally, sections were fixed for 5 min with 1% aqueous glutaraldehyde, washed twice for 5 min each time with deionized water, and then stained for 5 min with 4% aqueous uranyl acetate (SERVA Electrophoresis GmbH, Germany). After blotting with filter paper, the grids were air-dried and stored in a desiccator until examination under the microscope.

### Transmission electron microscopy and imaging of serial sections

The sections were imaged with a transmission electron microscope LEO 906 (Carl Zeiss Microscopy GmbH) at an acceleration voltage of 80 kV and recorded on sheet films (Kodak electron image film SO-163, Plano GmbH) with 1 s exposure times. The exposed negatives were removed from the microscope, sorted to racks and transferred within the racks for 4 min into a box containing the developer (Hans O. Mahn GmbH, Germany), for 30 s into water with acetic acid (10 ml of 10% acetic acid to 4 l of water), and for 6 min into the fixing solution (Hans O. Mahn GmbH). After 30 min rinsing with tap water and immersing into a wetting agent (Hans O. Mahn GmbH), the negatives were dried at 40 °C overnight. Finally, the negatives were scanned (Quato Intelli Scan 1600, software Silverfast Ai 6.5) at 600 dpi and edited using Adobe Photoshop CS6.

### 3D reconstruction of cells and analysis of Asp23 distribution

For 3D reconstructions, consecutive sections were aligned and outlines of cell structures were traced using Reconstruct^1^. Extent of cell wall blur (D) is calculated as x_2_-x_1_ with ${x}_{{\rm{n}}}=\sqrt{{r}_{n}^{2}-{s}_{n}^{2}}$. See Fig. 2b for assignment of variables. The traced cell structures were visualized as ‘traces’ or ‘boissonnat surface’. Immunofluorescence signals were marked with the ‘tracing’ tool and visualized as ‘trace slabs’. Gold particles in the micrographs of immunogold labeled thin-sections were marked with the ‘stamp’ tool and visualized as ‘spheres’ with a diameter of 40 nm, representing the possible space of the real position of the labeled epitope due to the linkage error.

A Perl script was developed for the extraction of 3D coordinates from image data that correspond to contours in XML files exported by Reconstruct. The script is freely available at https://github.com/KatharinaHoff/Reconstruct-Parser.

For the analysis of spatial Asp23 data, point pattern of gold particles were analyzed using Ripleys K function through its implementation in the R package spatstat^2^, version 1.59. This K function, capturing the inner geometry of the particle cloud, is contrasted to 95% pointwise simulated confidence bands which were obtained from different models: (i) complete spatial randomness, (ii) random distribution over a sphere as model for random distribution near to the cell wall, (iii) random distribution over a circle as model for one ring structure and (iv) random distribution over two parallel circles as model for two ring structures. Additionally, all points in the simulated point patterns were randomly jittered at a mean distance of 65 nm to reflect that the gold particles only approximate the location of protein molecules. Perspective plots of one simulation from each of the models and plots of the data K function together with the four confidence bands are provided as supplement.

### *S. aureus* mutant construction

*S. aureus* mutants were constructed according to the protocol of Müller *et al*. 2014^1^. Strains and plasmids used in this study are summarized in Supplementary Table S1. For the introduction of the A206K amino acid substitution by PCR into the cerulean gene fused to *asp23*, pMM033 was used as template with primers asp23cer_A206K_for; CTTAAGTACACAATCAAAGTTATCAAAAGATCCTAATG and asp23cer_A206K_rev; CTTTGATTGTGTACTTAAGTAATGATTATCAGGTAATAATACAGG.

### Fluorescence microscopy of intact cells

Fluorescence microscopy of cells immobilized on agarose pads was carried out on a Zeiss Axio Imager.M2 (Carl Zeiss Microscopy GmbH, Oberkochen, Germany) equipped with a 100×/NA 1.3 Neofluar objective as described earlier^1^. To detect cyan (Asp23-Cer) and red (Alexa Fluor 546 goat anti-rabbit IgG (H + L), Invitrogen Karlsruhe, Germany) fluorescence signals, filter set 47E and 63HE were used, respectively. For 3D reconstructions of the fluorescence signal from whole cells, bacteria were imaged with a Plan Apochromat 63×/NA 1.4 objective on a Zeiss LSM880 Airyscan confocal microscope (Carl Zeiss Microscopy GmbH) with xyz pixel size settings of 0.04 × 0.04 × 0.16 µm.

### Cloning, expression, purification Asp23-strep

For the expression of Asp23 proteins in *E. coli*, the *asp23* coding region of *S. aureus* was amplified by PCR using primers 20for; ATGGTAGGTCTCAAATGACTGTAGATAACAATAAAGCAAA and 22rev; ATGGTAGGTCTCAGCGCTTTGTAAACCTTGTCTTTCTTGGTTAT with chromosomal DNA from *S. aureus* COL as a template. The PCR product was digested with BsaI and cloned into pPR-IBA1 (IBA, Göttingen, Germany). The resulting plasmid was verified by DNA sequencing using primers 77for; TAATACGACTCACTATAGGG and 78rev; TAGTTATTGCTCAGCGGTGG.

Plasmids were transformed into *E. coli* BL21 (DE3) pLysS and cells were plated on Luria-Bertani (LB) plates containing chloramphenicol (25 µg ml^−1^) and ampicillin (100 µg ml^−1^). A few colonies were used to inoculate 20 ml of an LB overnight culture. The overnight culture was used to inoculate 1 l LB medium containing chloramphenicol and ampicillin to an OD_540_ of 0.05. At an OD_540_ of 0.4 expression of *asp23* was induced by addition of 1 mM IPTG. Cells were harvested two hours after induction by centrifugation (8,000 *g*, 10 min, 4 °C; Sorvall RC 6+ Centrifuge, Rotor F12-6 × 500 Lex; Thermo Fisher Scientific, Waltham, USA) and stored until further use at −20 °C.

For Asp23-strep purification, cells were disrupted by sonication (Sonopuls ultrasonic homogenizer HD3100, probe MS73; Bandelin elextronic GmbH, Berlin, Germany) in 20 ml buffer W (100 mM Tris, 150 mM NaCl, 5 mM MgCl_2_, pH 8.0). After 30 min centrifugation of the lysate at 8,000 g and 4 °C Megafuge 8 R Centrifuge; Thermo Fisher Scientific, Waltham, USA), the supernatant was filtered through a syringe attachment filter (0.45 µm pore size) and used for Strep-tag purification according to the manufacturer’s instructions (IBA, Göttingen, Germany).

### Negative staining of Asp23-strep

For transmission electron microscopy, Asp23-strep was subjected to negative staining to visualize protein complexes, directly after purification of the proteins. Fresh glow-discharged copper grids with a carbon-coated perforated pioloform film were put on droplets on or below the particle suspension (protein concentration 0.1–0.5 mg ml^−1^) for 8 min. After two washing steps with water, the grids were stained with 1% aqueous uranyl acetate for 30 seconds. Air-dried grids were examined with a transmission electron microscope LEO 906 (Zeiss, Oberkochen, Germany) at an acceleration voltage of 80 kV.

## Supplementary information

Supplemantary Information

Supplemantary Information 2

Supplemantary Information 3

Supplemantary Information 4

Supplemantary Information 5
