# Supplementary material for: Non-invasive and label-free 3D-visualization shows in vivo oligomerization of the staphylococcal alkaline shock protein 23 (Asp23)
Source: Sci Rep. 2020 Jan 10;10:125. doi: 10.1038/s41598-019-56907-9 (PMC6954212; doi:10.1038/s41598-019-56907-9)
Supplement: Supplementary file 5 — Supplemantary Information 5 [file 41598_2019_56907_MOESM5_ESM.docx]

**Supplementary Information**

**Non-invasive and label-free 3D-visualization shows *in vivo* oligomerization of the staphylococcal alkaline shock protein 23 (Asp23)**

Inga Petersen^1,4^, Rabea Schlüter^2^, Katharina J. Hoff^3,4^, Volkmar Liebscher^3^, Gert Bange^5^, Katharina Riedel^1,4^, Jan Pané-Farré^1,4,5#^

1) University of Greifswald, Institute of Microbiology, Felix-Hausdorff-Str. 8, 17489 Greifswald, Germany.

2) University of Greifswald, Imaging Center of the Department of Biology, Friedrich-Ludwig-Jahn-Str. 15, 17489 Greifswald, Germany.

3) University of Greifswald, Institute of Mathematics and Computer Science, Walther-Rathenau-Str. 47, 17489 Greifswald, Germany.

4) Center for Functional Genomics of Microbes, Felix-Hausdorff-Str. 8, 17489 Greifswald, Germany

5) Philipps-University Marburg, SYNMIKRO Research Center and Department of Chemistry, Hans-Meerwein-Strasse 6, C07, 35043 Marburg

#) Corresponding author:

Jan Pané-Farré

Philipps-University Marburg,

SYNMIKRO Research Center and Department of Chemistry,

Hans-Meerwein-Strasse 6, C07,

35043 Marburg, Germany

[jan.panefarre@chemie.uni-marburg.de](mailto:jan.panefarre@chemie.uni-marburg.de)

**Supplementary figures**

**
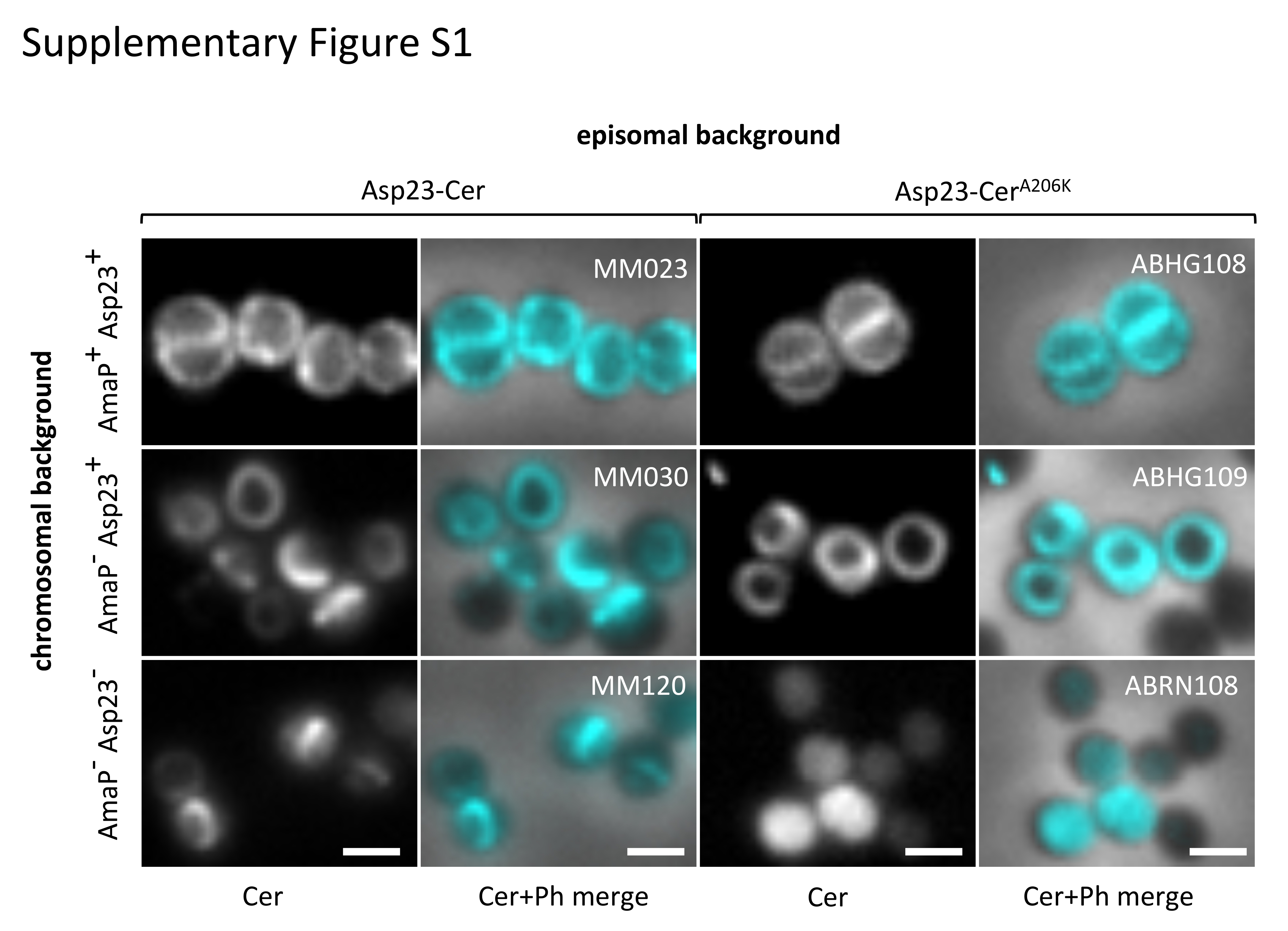
**

**Supplementary Figure S1. Asp23 localization is influenced by the fluorescence tag and AmaP availability.** Cells were cultivated with 1% xylose for episomal expression of fluorescently tagged Asp23. Untagged wild-type Asp23 appears to be necessary for ring-shaped oligomerization of C-terminal tagged Asp23 in *amaP-*deficient strains. In addition, self-interaction of Cerulean seems to promote Asp23-Cer oligomerization as the self-interaction-inhibiting point mutation A206K in the Cerulean tag results in diffuse Asp23-Cer^A206K^ distribution in strains lacking AmaP and wild type Asp23. Strains used are indicated in corresponding merged images. Scale bar 1 µm.


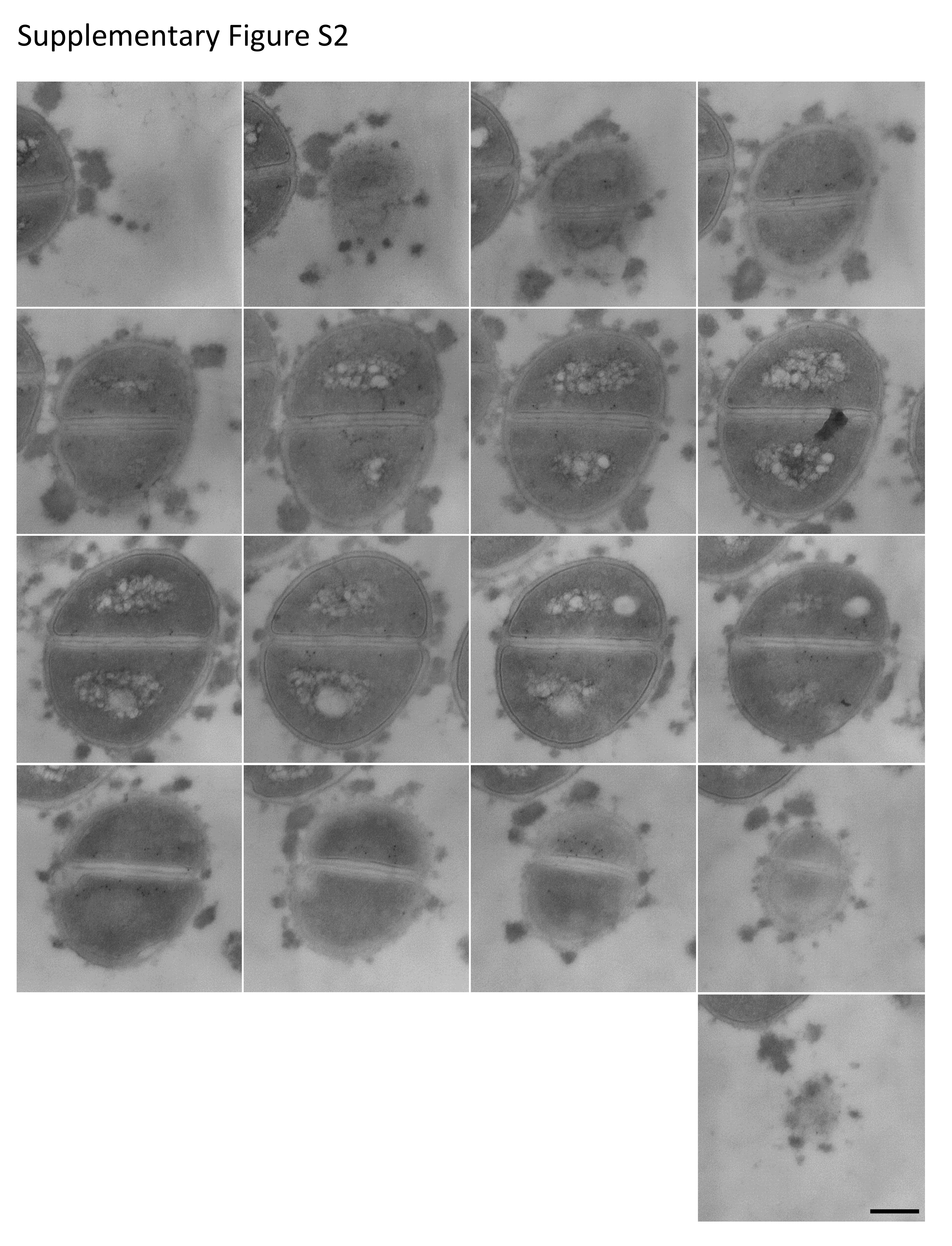


**Supplementary Figure S2.** Consecutive sections (50 nm thickness) of DT001 (*ΔamaP* Δ*spa*) with immunogold labeling (5 nm gold particles) of Asp23 used for 3D reconstruction Fig. 2f, (i). Scale bar 200 nm.


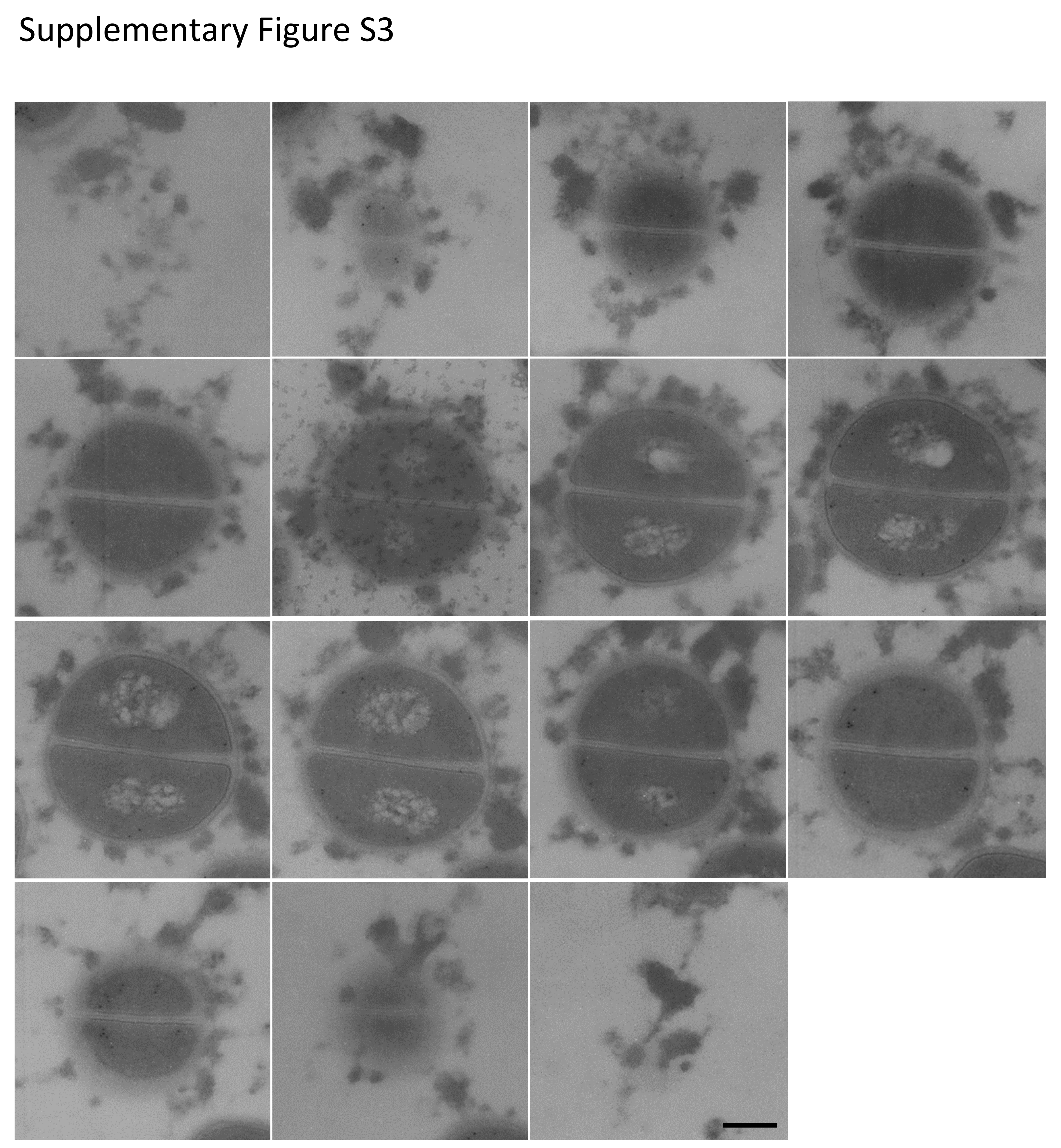


**Supplementary Figure S3.** Consecutive sections (50 nm thickness) of MM040 (HG001 Δ*spa*) with immunogold labeling (5 nm gold particles) of Asp23 used for 3D reconstruction shown in Fig. 2 f, (ii). Scale bar 200 nm.

**
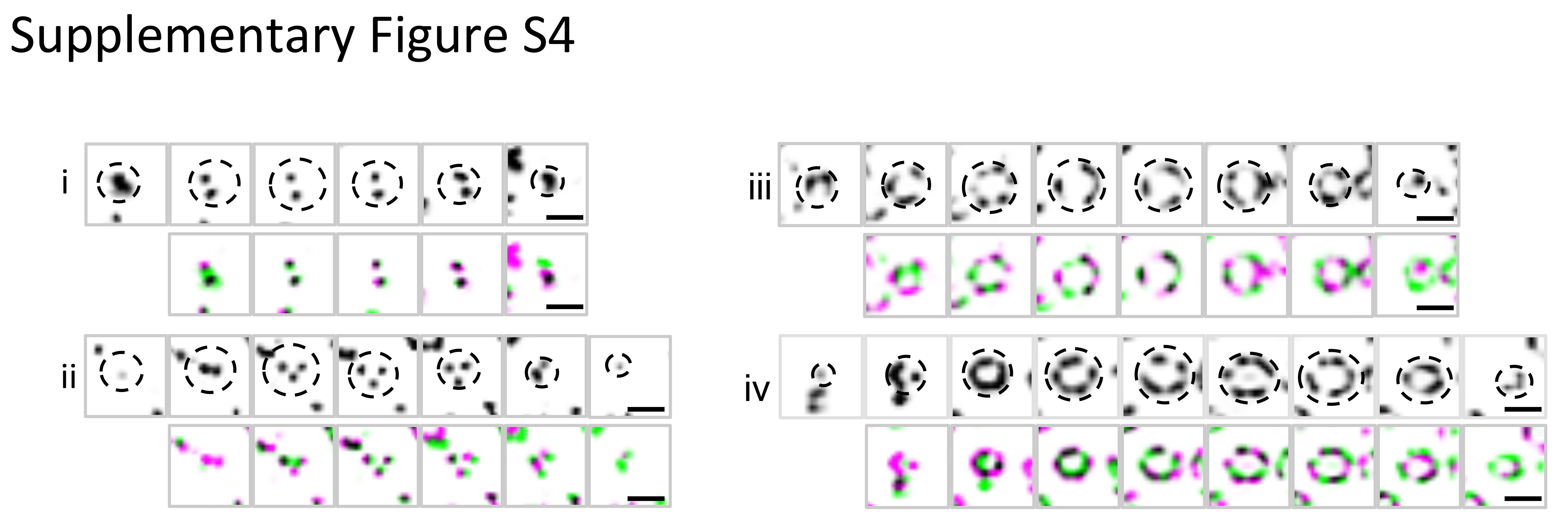
**

**Supplementary Figure S4.** Immunofluorescence signals for Asp23 in *amaP* mutant (i and ii) and wild type cells (iii and iv) used for 3D reconstructions shown in Fig. 2 e. Sections are 100 nm thick. Dotted line indicates estimated position of cells. Fluorescent signals in overlay images are indicated in green = preceding ection, magenta = subsequent section. Scale bar 1 µm.


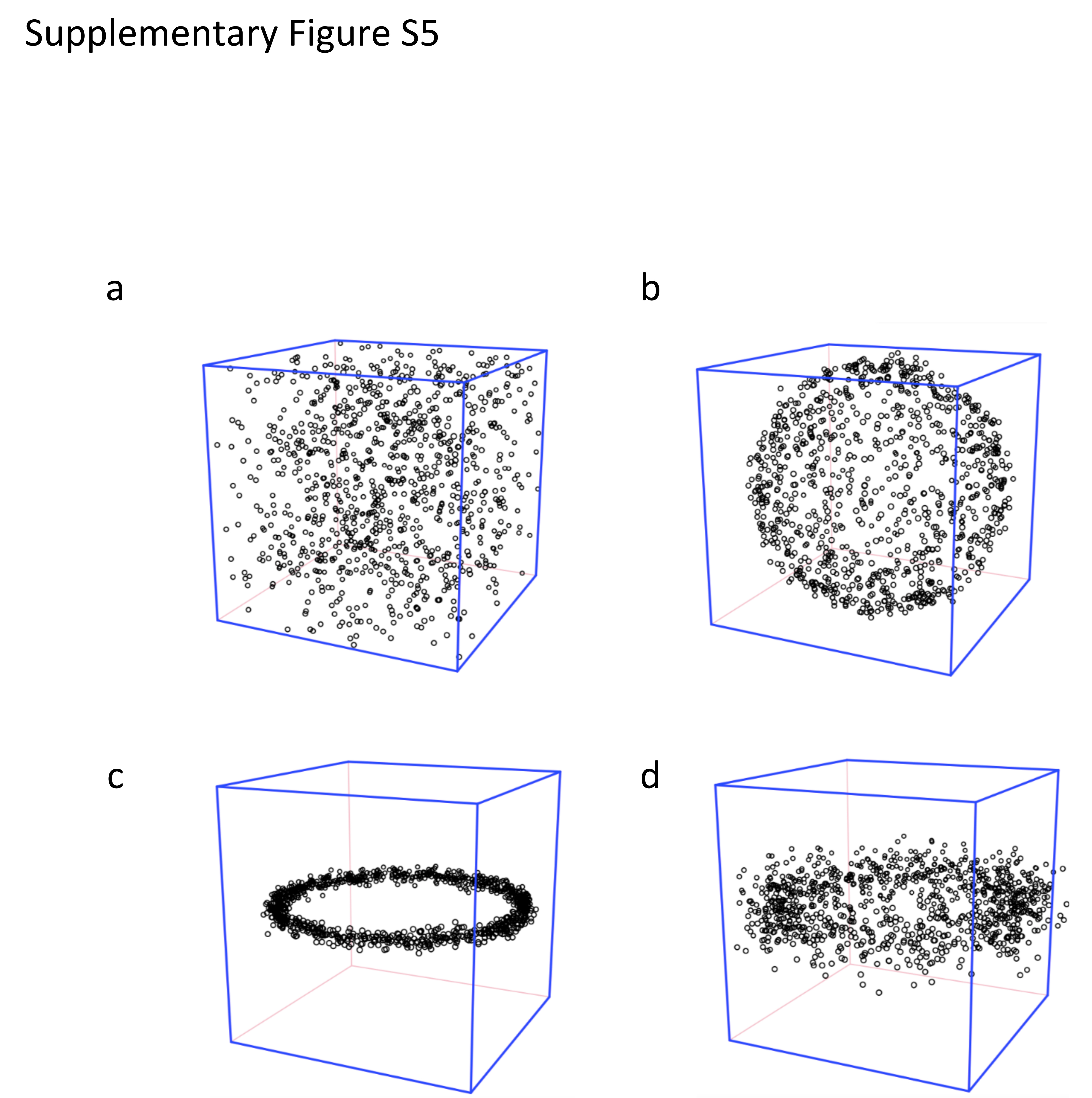


**Supplementary Figure S5.** Three-dimensional visualization of the different phantoms used in the simulations for interpreting the K-functions of the observed gold particle configurations: a) complete randomness b) random distribution on the surface of a sphere c) random distribution on a ring and a ring with d) quadruple jittering. The ring structure(s) are abstractions of the findings from widefield visualization of Cerulean-tagged Asp23.

**
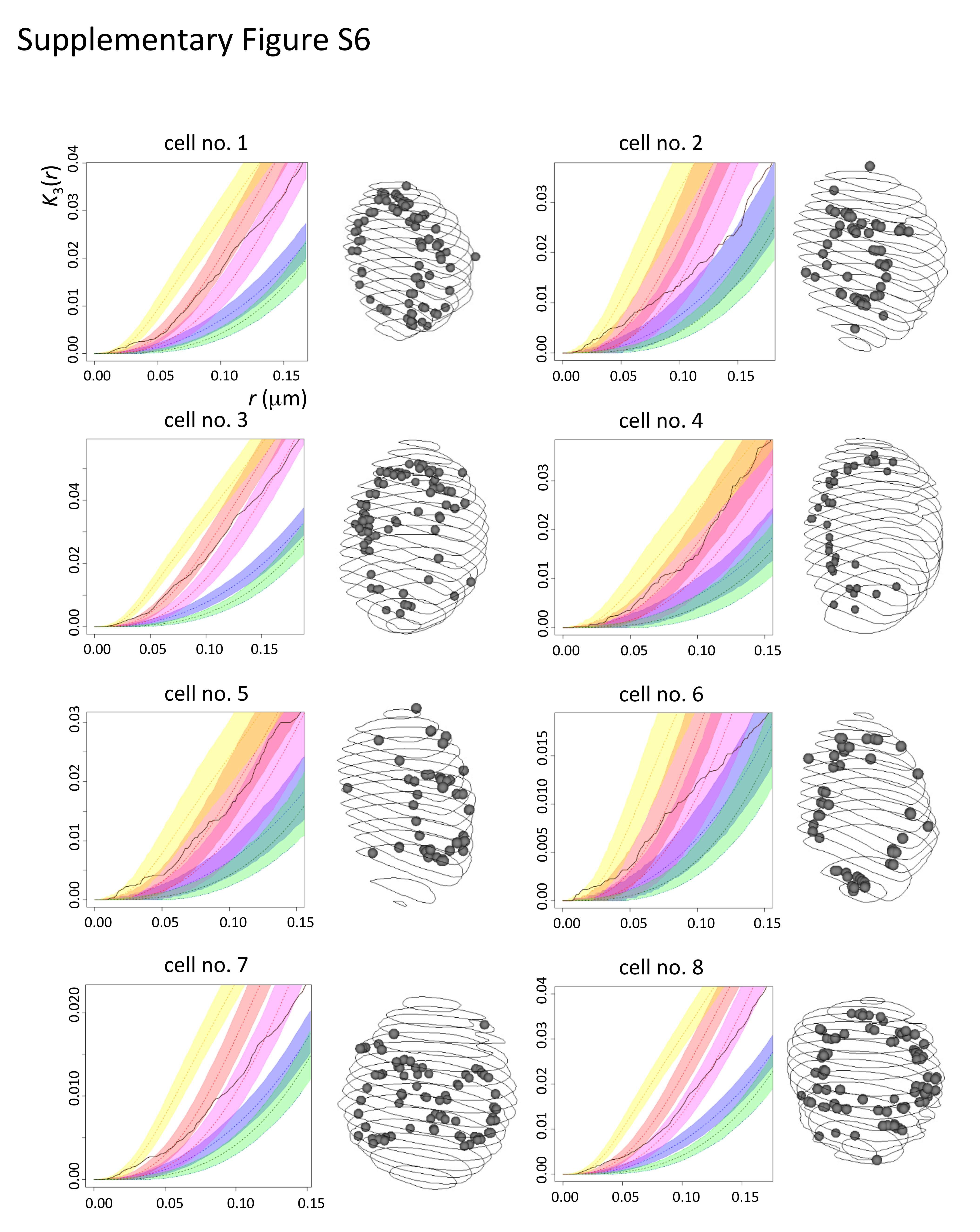
**

**Supplementary Figure S6.** K-functions for the distributions of gold particles in the mutant cells. Distributions are compared to simulated K-functions shown in supplementary figure S5: black: gold particle data; green: complete random distribution; blue: spherical distribution; yellow: single ring with ordinary jittering; pink: ring with double jittering; violet: ring with quadruple jittering. The shadowed areas provide pointwise 95% confidence bands using an envelope of the simulations. Data clearly lie outside the green and blue bands, disproving a random distribution inside the whole cell volume or near the cell wall. Nearness of the data K-function to the violet bands suggests an underlying ring structure. Corresponding 3D-rendered distribution of gold particles within cells is shown on the left side of each graph. For dividing cells the two related half spheres we analyzed individually.

**
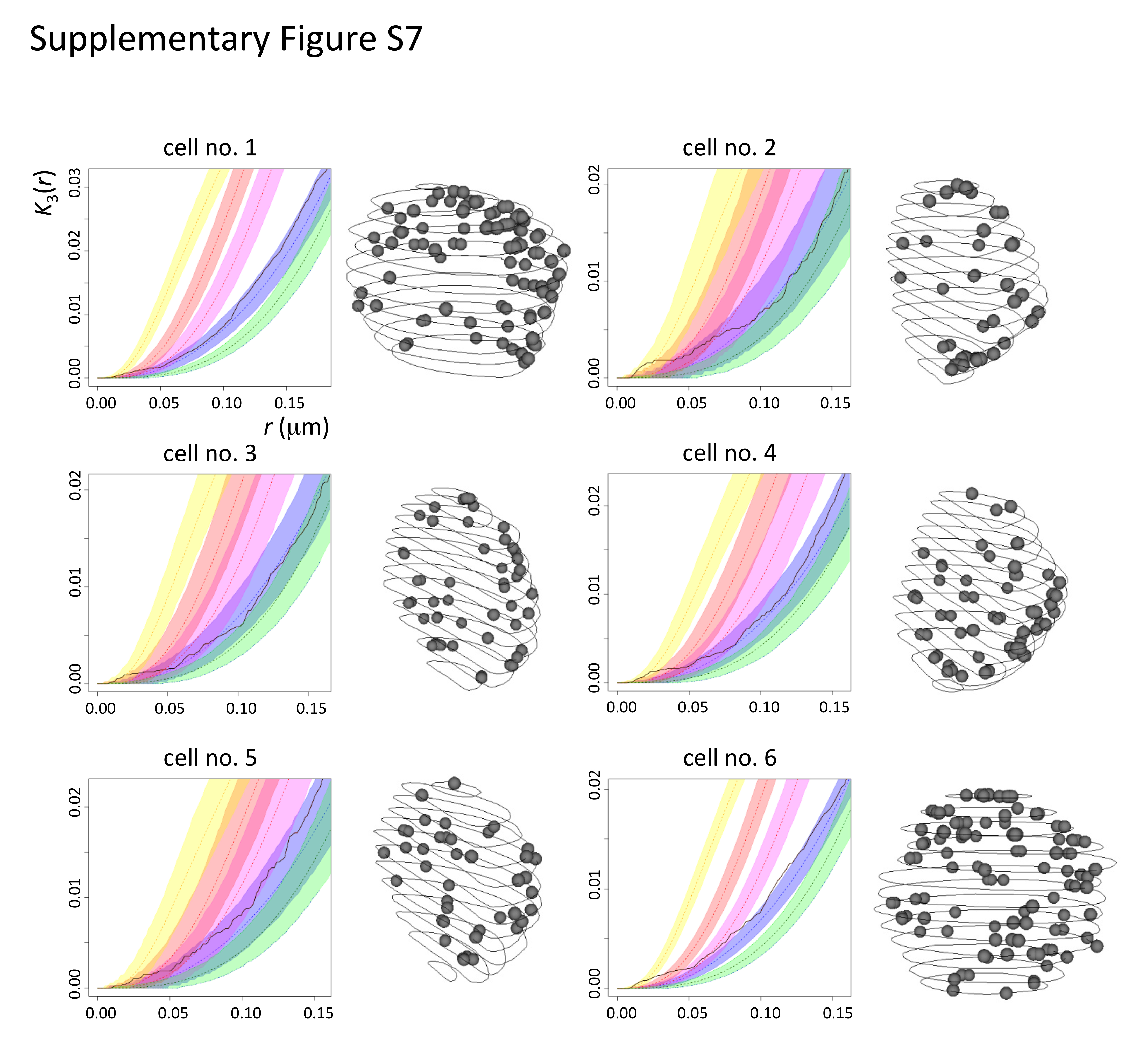
**

**Supplementary Figure S7.** K-functions for the distributions of gold particles in the mutant cells. Distributions are compared to simulated K-functions shown in supplementary figure S5: black: gold particle data; green: complete random distribution; blue: spherical distribution; yellow: single ring with ordinary jittering; pink: ring with double jittering; violet: ring with quadruple jittering. The shadowed areas provide pointwise 95% confidence bands using an envelope of the simulations. The K-functions of the data clearly lies outside the red, green and yellow bands supporting that particles are located near the cell surface, and are not randomly distributed within the cell volume. Corresponding 3D-rendered distribution of gold particles within cells is shown on the left side of each graph. For dividing cells the two related half spheres we analyzed individually.

**Legends to supplementary movies**

**Supplementary Movie S1.** 3D reconstruction of Asp23 from electron micrographs of immunogold labeled serial sections wild type *S. aureus* cells. Red spheres = Asp23, blue volume = DNA, grey lines = membrane.

**Supplementary Movie S2.** 3D reconstruction of Asp23 from electron micrographs of immunogold labeled serial sections of *S. aureus* *amaP* mutant cells. Red and orange spheres = Asp23, blue volume = DNA, grey lines = membrane.

**Supplementary Movie S3.** 3D reconstruction of Asp23 from micrographs of immunofluorescence labeled serial sections of wild type *S. aureus* cells. Orange volume = Asp23. The blue sphere was drawn to enhance visualization of Asp23 distribution.

**Supplementary Movie S4.** 3D reconstruction of Asp23 from micrographs of immunofluorescence labeled serial sections of *S. aureus* *amaP* mutant cells. Orange volume = Asp23.

**Supplementary Table S1. Strains and plasmids**

|  | | **Description** | **Source or suppl. reference** |
| --- | --- | --- | --- |
| ***Escherichia coli*** | | | |
| DH5α | | F-φ80d*lac*Z Δ(*lac*ZYA-*arg*F) U169 *deo*R*sup*E44Δ*lac*U169 (f80*lac*ZDM15) *hsd*R17 *rec*A1 *end*A1 (rk- mk+) *sup*E44*gyr*A96 *thi*-1 *gyr*A69 *rel*A1 | 1 |
| BL21 (DE3) pLysS | | F- *omp*T *hsd*S *gal* (rb- mb+) DE3(Sam7 Δnin5 *lac*UV5-T7 Gen1), *cat* | 2 |
| ***Staphylococcus aureus*** | | |  |
| SA113 Δ*spa* | | SA113 Δ*spa,* Em^r^ | C. Wolz, Tübingen |
| RN4220 | | restriction negative strain | 3 |
| MM120 | | RN4220 pMM033 | 4 |
| ABRN108 | | RN4220 pAB106 | this study |
| HG001 | | RN1 derivative, *rsbU* repaired, *tca*R | 5 |
| MM005 | | HG001 Δ*asp23* | 4 |
| MM040 | | HG001 Δ*spa*, Em^r^ | 4 |
| MM039 | | MM005 Δ*spa*, Em^r^ | 4 |
| MM023 | | HG001 pMM033 | 4 |
| ABHG108 | | HG001 pAB106 | this study |
| MM007 | | HG001 Δ*amaP* | 4 |
| DT001 | | HG001 Δ*amaP* Δ*spa*, Em^r^ | this study |
| MM030 | | MM007 pMM033 | 4 |
| ABHG109 | | MM007 pAB106 | this study |
| **Bacteriophages** | | | |
| *S. aureus* phage 80 |  | | 6 |
| **Plasmids** |  | |  |
| pAB106 | pRB473-*xyl*R-*asp23*-*cer*A206K, *cat* | | this study |
| pMM033 | pRB473-xylR-*asp23*-*cer*, *cat* | | 4 |
| pMM003 | pPR-IBA1-*asp23*, *bla* | | 4 |

**Supplementary references**

1. Hanahan, D. Studies on transformation of Escherichia coli with plasmids. *J. Mol. Biol.* **166**, 557–580 (1983).

2. Studier, F. W. & Moffatt, B. A. Use of bacteriophage T7 RNA polymerase to direct selective high-level expression of cloned genes. *J. Mol. Biol.* **189**, 113–130 (1986).

3. Kreiswirth, B. N. *et al.* The toxic shock syndrome exotoxin structural gene is not detectably transmitted by a prophage. *Nature* **305**, 709–712 (1983).

4. Müller, M. *et al.* Deletion of membrane-associated Asp23 leads to upregulation of cell wall stress genes in Staphylococcus aureus. *Mol. Microbiol.* **93**, 1259–1268 (2014).

5. Herbert, S. *et al.* Repair of global regulators in Staphylococcus aureus 8325 and comparative analysis with other clinical isolates. *Infect. Immun.* **78**, 2877–2889 (2010).

6. ROSENBLUM, E. D. & TYRONE, S. SEROLOGY, DENSITY, AND MORPHOLOGY OF STAPHYLOCOCCAL PHAGES. *J. Bacteriol.* **88**, 1737–1742 (1964).
